# Hemodynamic Changes Caused by Flow Diverters in Rabbit Aneurysm Models: Comparison of Virtual and Realistic FD Deployments Based on Micro-CT Reconstruction

**DOI:** 10.1371/journal.pone.0066072

**Published:** 2013-06-18

**Authors:** Jinyu Xu, Benqiang Deng, Yibin Fang, Ying Yu, Jiyong Cheng, Shengzhang Wang, Kuizhong Wang, Jian-Min Liu, Qinghai Huang

**Affiliations:** 1 Department of Neurosurgery, Changhai Hospital, Second Military Medical University, Shanghai, China; 2 Department of Neurology, Changhai Hospital, Second Military Medical University, Shanghai, China; 3 Department of Mechanics and Engineering Science, Fudan University, Shanghai, China; University of Adelaide, Australia

## Abstract

Adjusting hemodynamics via flow diverter (FD) implantation is emerging as a novel method of treating cerebral aneurysms. However, most previous FD-related hemodynamic studies were based on virtual FD deployment, which may produce different hemodynamic outcomes than realistic (*in vivo*) FD deployment. We compared hemodynamics between virtual FD and realistic FD deployments in rabbit aneurysm models using computational fluid dynamics (CFD) simulations. FDs were implanted for aneurysms in 14 rabbits. Vascular models based on rabbit-specific angiograms were reconstructed for CFD studies. Real FD configurations were reconstructed based on micro-CT scans after sacrifice, while virtual FD configurations were constructed with SolidWorks software. Hemodynamic parameters before and after FD deployment were analyzed. According to the metal coverage (MC) of implanted FDs calculated based on micro-CT reconstruction, 14 rabbits were divided into two groups (A, MC >35%; B, MC <35%). Normalized mean wall shear stress (WSS), relative residence time (RRT), inflow velocity, and inflow volume in Group A were significantly different (P<0.05) from virtual FD deployment, but pressure was not (P>0.05). The normalized mean WSS in Group A after realistic FD implantation was significantly lower than that of Group B. All parameters in Group B exhibited no significant difference between realistic and virtual FDs. This study confirmed MC-correlated differences in hemodynamic parameters between realistic and virtual FD deployment.

## Introduction

Hemodynamic modification is one objective of aneurysm treatment, as hemodynamic factors are commonly believed to play an important role in the pathogenesis, progress, and rupture of cerebral aneurysms. [1], [2] The application of an endovascular stent allows many complex aneurysms to be treated with intervention therapy. [3] The purpose of a stent placed in the parent artery is not only to protect the parent artery from occlusion, but also to act as flow diversion. [4]–[7] This hemodynamic effect was further confirmed in some clinical studies, which showed that stent-assisted embolization had a higher long-term healing rate than coil embolization. [8] The use of flow diverters (FDs) is an emerging paradigm for treating traditionally difficult cerebral aneurysms, such as wide-necked, large or fusiform aneurysms, [9]–[11] by using a fine mesh to divert flow away from the aneurysm sac. These stent-like devices are designed to divert blood flow along the normal anatomical course of the vessel and away from the aneurysm dome. Comparing stent-induced and FD-induced changes in flow over the aneurysm neck showed that FDs reduced the flow velocities more significantly than stents; however, the reduction was dependent upon design and appropriate positioning. [12] Although an increasingly number of FDs have been applied in intracranial aneurysms as a novel therapy, the variability of its clinical outcomes emphasizes a need to investigate the hemodynamic effects of FDs on patient-specific aneurysms. Most, if not all, previous studies were based on virtual stent deployment methods, including but not limited to the adaptive grid embedding technique, porous medium method, fast virtual stenting method, and so on. One major limitation of these techniques is that they assume ideally deployed stents with a high degree of symmetry and uniformity, which contrasts with the significant changes in porosity and mesh hole shape known to occur during FD implantation. [13] Previous studies also proved that the hemodynamics of aneurysms could be observably affected by the porosity and mesh hole shape of FDs. Whether the difference in clinical outcomes is related to the variable configuration of the deployed FD remains to be confirmed, which has caused uncertainty regarding the accuracy of FD treatment. The variable configurations may also be the key points causing the different outcomes obtained when analyzing the stent-related hemodynamic parameters.

Accurately representing the realistic configuration of deployed FDs in vessel lumens *in vivo* remains technically challenging. Although micro-CT can be used to reconstruct the realistically deployed states of the stent, [14], [15] it is impossible to reconstruct stents with micro-CT in patients. Ma et al. [16] developed a finite element analysis (FEA) based workflow to simulate the mechanical deployment of FDs in patient-specific aneurysms, which demonstrated the mechanical modeling of braided FD stent deployment in cerebral vasculature to produce realistic deployment configuration. The cardiac and vortical flow analysis methods and the fluid-structure interaction (FSI) analysis may also provide insights into the blood flow within the aneurysm and the interaction of artery and blood. [17]–[20] However, whether this *in vitro* research reflects the *in vivo* situation needs to be further explored. And further studies are needed to evaluate the safety of these stent-like devices by using a systematic order of approach. [21] In light of the increasing clinical need for accurate CFD analysis of FD treatment and the inability of current numerical methods to produce realistically deployed FD geometries, this study was designed to investigate the hemodynamic effects of different FDs in rabbit aneurysm models with virtually deployed FDs reconstructed using SolidWorks software and realistically deployed FDs reconstructed based on micro-CT images.

## Materials and Methods

This study was approved by the institutional animal care and use committee of Second Military Medical University. The FD (first-generation TUBRIDGE embolization device; MicroPort Medical (Shanghai) Co. Ltd., China) used in this experiment is composed of 32 nickel–titanium alloy strands, including two parallel radiopaque struts containing platinum (diameter = 0.05 mm). The virtual FD, built for comparison, has the same shape parameters.

### Animal Experiments

Fourteen wide-necked aneurysms were induced using elastase to digest the right common carotid artery of healthy adult New Zealand white rabbits of both sexes (body weight, 3–4 kg), as previously described [22]. FD treatment, angiographic follow-ups and segmentation of parent vessels including FDs were performed as previously described. [4] To confirm the exact position of the aneurysm neck, we marked it with steel-wire needles.

**Figure 1:**
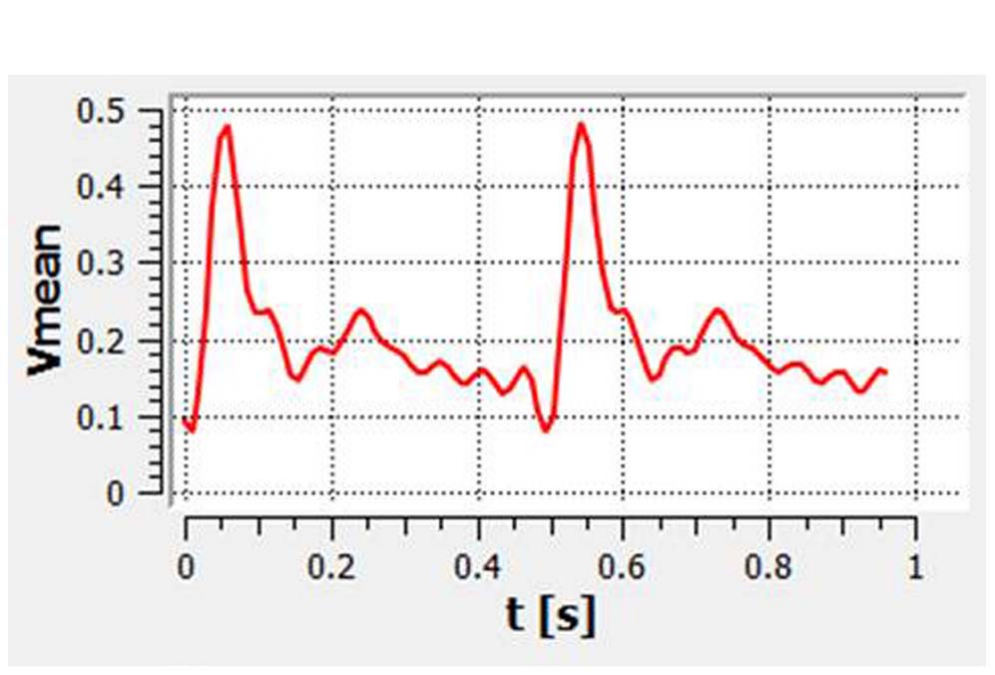
The flow velocity waveform based on transthoracic duplex Doppler examination.

The pulsatile velocity waveform was obtained by transthoracic duplex Doppler in the innominate artery of one healthy rabbit. We then digitized the Doppler spectrum envelope to obtain the blood flow velocity waveform (Figure 1) in a whole cardiac cycle using Matlab 7.0 software (MathWorks, Natick, Massachusetts).

### Rabbit-specific Aneurysm Modeling

Images of 14 rabbit-specific aneurysm models were obtained from Integris Allura Flat digital subtraction angiography (Philips Healthcare, Best, Netherlands) and then reconstructed using the Philips Allura FD20 workstation to produce a virtual reality modeling language (VRML) format. The VRML files were converted to a stereolithography (STL) format using 3DMAX8.0 (Autodesk USA). Subsequently, image models were segmented to remove unwanted structures, while cerebral vessels and aneurysms were kept for analysis. The surface of the segmented models was smoothed by GEOMAGIC STUDIO 9.0 software (Geomagic USA).

### 3D FD Micro-CT Reconstruction and Virtual FD Construction

Three months after FD deployment, the rabbits were sacrificed by intravenous injection of sodium pentobarbital (100 mg/kg). To confirm the exact position of the aneurysm neck, the entire segment of the parent vessel, including the FD, was marked with steel-wire needles and then dissected. The tissue including the FD was pressure-fixed with 10% formalin under approximately 100 cm of water pressure for approximately 4 hours and then stored in the same solution for at least 24 hours. All embedded samples, which were marked by steel-wire needles in the aneurysm neck, were scanned using micro-CT (GE eXplore Locus SP) with a 17-μm pixel size. The scanned CT data of FDs were processed with Mimics (v. 10.01, Materialise, Leuven, Belgium) to reconstruct realistic 3D FD geometries. The strut diameter was modified to 0.05 mm by adjusting the image threshold. Virtual FDs were constructed with SolidWorks 2010 (Dassault Systemes, Concord, Massachusetts). We calculated the metal coverage of each FD according to the measurement of the reconstructive shape by micro-CT, as previously described [4].

### Meshing and Flow Modeling

The FDs were fitted into the parent vessel lumen across the aneurysm neck using GEOMAGIC STUDIO 9.0 software (Geomagic USA). And the realistic FDs were fitted according to the steel-wire marker. The FD-implanted aneurysms were merged in ICEM CFD 11.0 (ANSYS, Lebanon, New Hampshire) to create volume grids for fluid dynamics computation. Each model was meshed to create 2.23 to 3.75 million finite volume tetrahedral elements and wall prism elements (for accurate boundary layer resolution), with 4.72×10^3^ to 1.41×10^4^ elements per cubic millimeter.

We treated blood as a Newtonian fluid. The density and dynamic viscosity of blood were specified as ρ = 1050 kg/m^3^ and μ = 0.0035 Pa·s, respectively. The governing equation underlying the calculation was the Navier-Stokes formulation, with an assumption of laminar and incompressible blood flow. The vessel was assumed to be rigid with no-slip boundary conditions. The inlet was imposed by pulsatile velocity profile measured from ultrasound Doppler as described before. The outlet was modeled as opening boundary condition with zero static pressure. The simulation was performed by CFX 11.0 (ANSYS). We discretized the whole cardiac cycle of 0.48 seconds by a time-step of 0.001 seconds for numeric simulation. Three cardiac cycles were simulated to ensure a full periodicity, and the last cycle was taken as output. The aneurysm geometries were isolated from their parent arteries for subsequent data analysis. We then post-processed and visualized the results of these simulations with CFX.

### Hemodynamic Parameter Calculation

We calculated the following hemodynamic parameters before and after the FD implantation: wall shear stress (WSS), pressure, relative residence time (RRT), inflow velocity, and inflow volume. WSS (already time-averaged, as in Eq. 1.) was averaged over the sac area (the entire luminal surface of the aneurysm sac). In this study, the WSS distributions were normalized by the average parent vessel WSS in the same rabbit to allow comparison among different models. [23] RRT, a combination of WSS and Oscillatory Shear Index (OSI), reflects the residence time of blood near the wall. [24] Thus, a new metric termed RRT was defined to quantify the state of disturbed flow: [25]

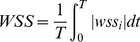
(1)

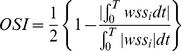
(2)

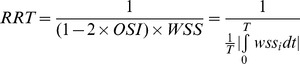
(3)where wss_i_ is the instantaneous WSS vector and T is the duration of the cycle.

The mean inflow velocity and inflow volume after FD implantation were calculated at the aneurysm neck at peak systole (t = 0.06 s). We also observed the inflow stream of the aneurysm sac by velocity magnitudes on a cut plane and streamlines after FD implantation.

### Statistical Analysis

The means and standard deviations (SDs) of all hemodynamic parameters were calculated for Groups A and B, and the data were expressed as the mean ± SD. Differences between groups were analyzed by the Mann-Whitney U test. Differences between realistic FD and virtual FD deployment were analyzed using a Wilcoxon signed-rank test. A P value <0.05 was regarded as statistically significant, and all tests were 2-sided. Statistical analyses were performed using Microsoft Excel 2003, Matlab 7.0 (Math Works, Natick, Massachusetts) and SPSS17.0 (SPSS Inc., Chicago, Illinois).

## Results

In total, 14 rabbit-specific aneurysm models were constructed. According to the real metal coverage (MC) of the implanted FDs based on micro-CT reconstruction, the 14 aneurysm models were divided into Group A (n = 8, MC >35%) and Group B (n = 6, MC <35%). In Group A, the MC ranged from 35.98% to 49.72% with a mean value of 43.96%. In Group B, the MC ranged from 21.19% to 29.45% with a mean value of 24.97%. The MC of the implanted virtual FDs ranged from 29.42% to 31.06% with a mean value of 30.02%. Baseline data, including average neck diameter, maximum perpendicular height of the aneurysm, aspect ratio (AR) value, and the hemodynamic parameters before the FD implantation, showed no significant difference between Group A and Group B (P>0.05).

**Figure 2:**
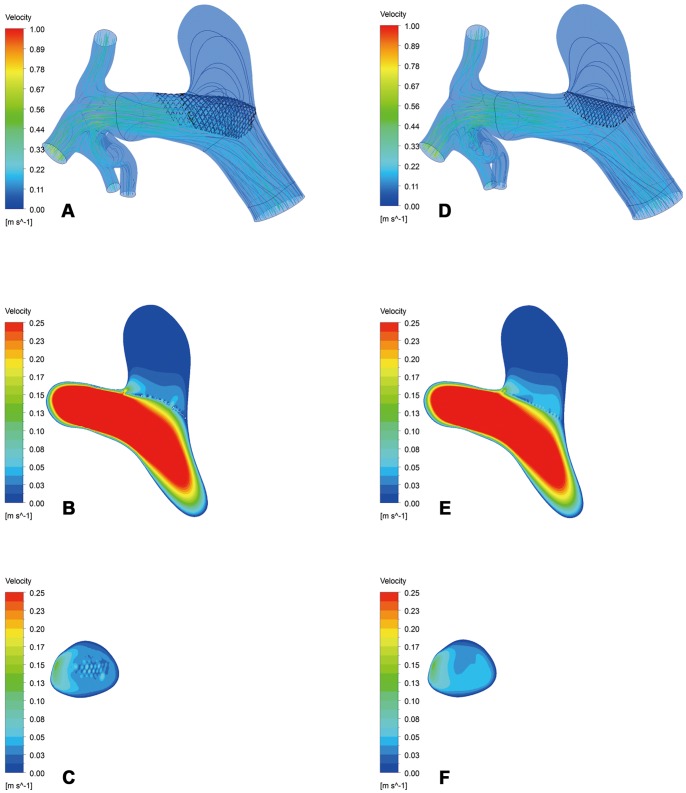
Inflow stream of the aneurysm sac on the cut plane and streamlines in Group B. A–C, The realistic FD. D–F, The virtual FD.

**Figure 3:**
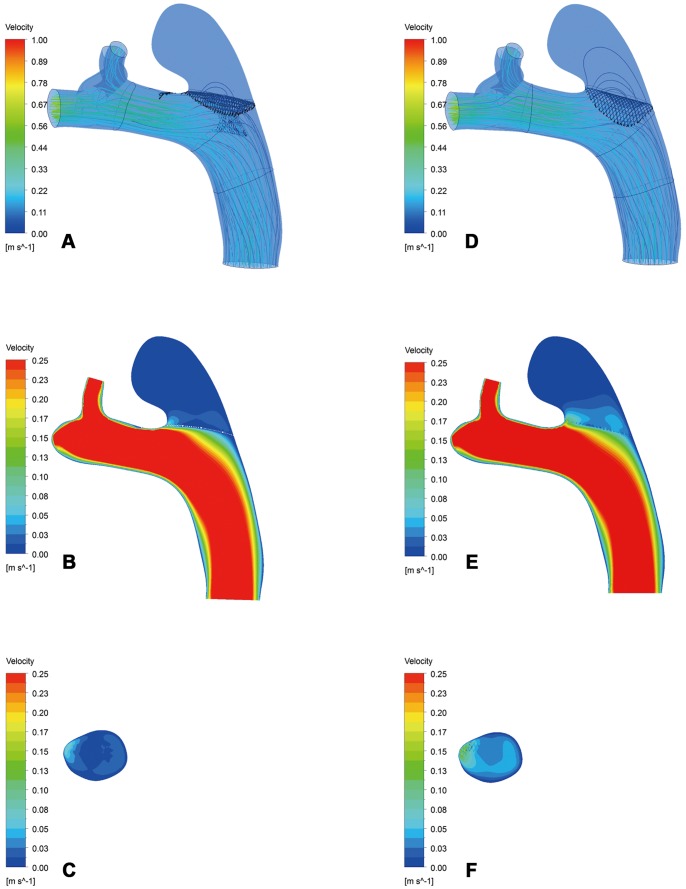
Inflow stream of the aneurysm sac on the cut plane and streamlines in Group A. A–C, The realistic FD. D–F, The virtual FD.

**Figure 4:**
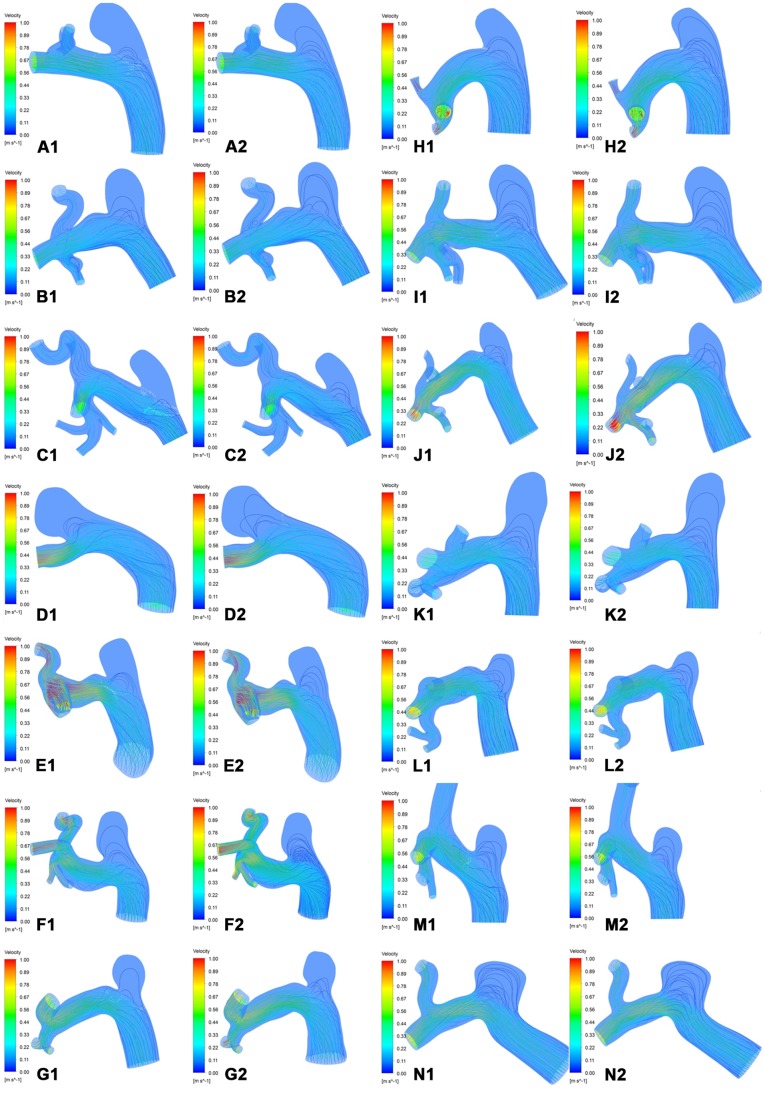
Inflow streamlines of the aneurysm sac. A–H, Group A. I–N, Group B. 1, The realistic FD. 2, The virtual FD.

As shown in Figure 2, the inflow stream of the aneurysm sac on the cut plane and the streamlines of the realistic and virtual FDs were almost the same in Group B. In contrast, Group A showed fewer streamlines of the realistic FD compared to the virtual FD, and the velocity of inflow stream at the aneurysm sac on the cut plane of the realistic FD was weaker than that of the virtual FD(Figure 3). The comparison of inflow streamlines of the 14 rabbit-specific aneurysm models (realistic and virtual FDs) are presented in Figure 4.

The means and SDs for each parameter are presented in Table 1. The hemodynamic parameters after the realistic deployment of FDs was reconstructed on micro-CT images were compared between Group A and Group B. The normalized mean WSS of the aneurysm sac after FD deployment in Group A was significantly lower than that in Group B. Additionally, the mean inflow velocity in Group A was slightly lower than that in Group B, although the difference was not significant (P = 0.059). There was no significant difference in pressure, RRT or inflow volume between the two groups. With virtual FD deployment, there was no significant difference in normalized mean WSS, pressure, RRT, inflow velocity or inflow volume between the two groups.

**Table 1:** Hemodynamic Differences between Realistic and Virtual Flow Diverters within and between Groups.

	Group A(n = 8)			Group B(n = 6)			RealisticFD	VirtualFD
Parameter	Realistic FD(mean ± SD)	Virtual FD(mean ± SD)	P value	Realistic FD(mean ± SD)	Virtual FD(mean ± SD)	P value	P value*	P value#
**WSS**	0.11±0.05	0.19±0.09	0.018	0.24±0.14	0.25±0.16	0.917	0.008	0.414
**Pressure, Pa**	101.18±67.94	99.72±62.34	0.612	123.79±68.58	121.57±69.28	0.116	0.534	0.573
**RRT**	4381.29±6723.75	1220.85±1788.29	0.028	904.71±1129.32	2223.58±3809.15	0.753	0.234	0.950
**Inflow velocity, m/s**	0.03±0.02	0.05±0.03	0.012	0.05±0.03	0.04±0.02	0.463	0.059	0.950
**Inflow volume, m^3^/s**	3.03×10^−7^±3.69×10^−7^	3.95×10^−7^±3.52×10^−7^	0.012	4.39×10^−7^±1.77×10^−7^	3.20×10^−7^±1.59×10^−7^	0.345	0.142	0.950

WSS stands for wall shear stress, and RRT stands for relative residence time.

* Statistical analysis of all parameters examined between two groups with realistic FD.

# Statistical analysis of all parameters examined between two groups with virtual FD.

In Group A, the normalized mean WSS of the aneurysm sac with realistic FD was lower than that with virtual FD (P = 0.018). There was also a significant difference in RRT, inflow velocity, and inflow volume between the realistic and virtual FDs (P<0.05) but no difference in pressure. In Group B, there was no significant difference between realistic and virtual FD deployment in any hemodynamic parameters.

## Discussion

In this study, we analyzed hemodynamic changes in rabbit aneurysm models by comparing micro-CT reconstruction-based realistic FDs with virtual FDs. Among the parameters, the normalized mean WSS, RRT, inflow velocity, and inflow volume exhibited significant differences between realistic and virtual FDs in the high realistic MC group. Normalized mean WSS after realistic FD deployment in the high MC group was also significantly lower than that in the low MC group.

Investigating the hemodynamic effects of fully deployed FDs requires reproducible FDs. A 3D stent model might be geometrically bent and fitted into the parent artery of the aneurysm [26], [27], but the stent would be unrealistically hanging in the lumen without expansion or apposition at the artery wall. A step-up method, namely adaptive grid embedding technique, was introduced by Cebral and Löhner [28] and further studied by Appanaboyina et al. [29]. This method produced a more appealing deployed geometry with wall apposition by accounting for stent expansion but failed to model stent mechanics and account for mechanical responses involved in stent deformation. The use of a porous medium layer with flow resistance equal to that of the FD [30] and the Fast Virtual Stenting (FVS) method [31] may reduce the number of calculation elements and any entailing computational costs. However, they do not reflect the details of the mechanical behavior of the stent. Although Ma et al. [16] simulated the mechanical deployment of FDs in patient-specific aneurysms by using a finite element analysis (FEA) based workﬂow, the simulations still needed to be further demonstrated. No previously described method is capable of producing mechanically and realistically deformed stent geometries in realistic vessel lumens. Although micro-CT can be used to reconstruct the realistically deployed states of the stent, there are no previously published reports regarding micro-CT-reconstructed FD, and its accuracy *in vivo* remains to be further confirmed. Based on micro-CT scans in rabbits, we reproduced the realistic FD *in vivo* and calculated its MC for a CFD study. To compare the virtual FD with the realistic FD reconstructed based on micro-CT, we adopted the 3D virtual FD model, as it not only reflected the structure of the FD but also required less time. As shown by our results, both types of FD deployment had similar hemodynamic results in Group B when the metal coverage was approximate. These results suggest the feasibility of our method of virtual FD deployment.

The hemodynamic efficiency of a stent is related to several parameters, including strut shapes, porosity, quantity of stents, mesh hole shapes, metal coverage, and so on. Kim et al [32] studied the effects of stent strut shape and porosity on hemodynamic flow inside an aneurysm using the lattice Boltzmann method (LBM) for numerical analysis and found that a rectangular stent was optimal and decreased the magnitude of velocity in the aneurysm sac by 89.25% in the 2D model and by 53.92% in the 3D model. Ionita et al. [33] proposed an asymmetric vascular FD with non-uniform porosity to treat aneurysms in animals and obtained results indicating that FD porosity significantly influenced the process of aneurysm occlusion. For the one-, two-, and three-layer stented models, total inflows to the aneurysm were reduced to approximately 75%, 37.5%, and 25%, respectively, in comparison to an unstented model [34]. Dorn et al. [12] compared the flow changes caused by three different stents (Solitaire, Silk, Phenox flow diverter) over the aneurysm neck. Their results showed that the flow diverters reduced the flow velocities by more than 50%, depending both on stent design and appropriate positioning, while the flow reduction was negligible with the Solitaire stent. Experimental studies have been conducted to investigate the effect of the stent blocking ratio *C*_α_ on the inflow into the aneurysm using particle tracking velocimetry (PTV) measurements and flow visualization of pulsatile flow fields in a stented cerebrovascular lateral aneurysm model. These studies showed that *C*_α_ = 75% resulted in the best attenuation of the risk of aneurysmal rupture and promotion of intra-aneurysmal thrombus. [35] Other experimental studies showed that the geometric configurations of stents affect vascular injury and neointimal hyperplasia, while surface material plays a greater role in thrombosis. [36] It has also been suggested that the inclusion of gaps between wire pitches reduces the neointimal thickness within stents and prevents the obstruction of arterial side branches [37] In our previous report [4], by reconstructing from micro-CT scanning files, we calculated the actual metal coverage at the neck of the FD. The results showed that aneurysm occlusion was positively correlated with the local metal coverage of the stent at the neck. And in current research, when deployed with realistic FDs, the normalized mean WSS and mean inflow velocity of the aneurysm sac in the high MC group was lower than that in the low MC group. These results further confirmed that the hemodynamic changes were related to the metal coverage of the FD at the neck and further emphasized the importance of accurately representing the FD.

Certainly, the results of computational fluid dynamic studies can be affected not only by the methods of FD deployment but also by specific factors such as boundary conditions and the morphology of the aneurysm and the parent artery, as the morphology is as important as hemodynamics in discriminating aneurysm rupture status. [2] The flow in an aneurysm may introduce vortex shedding, as well as interactions between blood and the vessel wall, where the aneurysm sac is irregular. [38], [39] Although differences in the normalized mean WSS and mean inflow velocity were observed between the high and low MC groups, the RRT and inflow volume exhibited no differences, possibly due to the influence of aneurysm morphology and the inherent correlation of RRT with both WSS and OSI. [24] To exclude the effects of aneurysmal and vascular morphology on hemodynamic differences, we further compared the hemodynamics after realistic and virtual FD deployment in the same aneurysm models. In Group A, significant differences in normalized mean WSS, RRT, inflow velocity, and inflow volume were observed between the realistic and virtual FDs. RRT is an additional hemodynamic factor that describes the slow flow motion near the aneurysm wall and reflects the residence time of blood near the wall. [24], [25] The difference in RRT between realistic FDs and virtual FDs may be due to the metal coverage of the FDs. As reported by Seong et al. [6], the FD with 35% MC could predict an angiographic aneurysm occlusion greater than 95%. We chose 35% MC as the grouping criterion for this reason. In contrast, in Group B, realistic and virtual FDs exhibited similar MC values, no parameters showed significant difference.

Although the changes of intra-aneurysmal pressure after FD deployment were disputed, we studied the changes of pressure after the deployment of realistic FDs with different MC values and found no significant pressure changes after FD deployment. Schneiders et al [40] measured intra-aneurysmal pressure before, during, and after placement of a flow-diverting stent using a dual-sensor guidewire and found that the pressure inside the aneurysm momentarily decreased during placement but was restored to baseline values within minutes. However, our CFD study was based on the steady state of blood flow after FD deployment as we simulated three cardiac cycles and outputted the last cycle. These might be the reasons for the unchanged pressure. The inflow stream of the aneurysm sac on the cut plane and the streamlines of the realistic and virtual FDs were almost the same in Group B but not in Group A. This may be due to the different MC values between realistic and virtual FDs and further demonstrates the effect of metal coverage on hemodynamic parameters.

### Limitations

The insight derived from computational simulation methodologies provides us with an improved understanding of this field. However, there are some limitations to this study. First, a single flow waveform obtained from a healthy animal subject was used in all CFD calculations, which might affect the validity of our results. A rabbit-specific condition should be used in the future to obtain more accurate results. Second, this study confirmed the different effects of realistic and virtual FDs on hemodynamics. However, the sample size could be considered to be slightly small. Finally, although we used micro-CT to reproduce the real configurations of flow diverters in parent vessels, the limited resolution of the micro-CT might cause slight distortions.

### Conclusion

This study compared the real structural configurations of fully deployed FDs *in vivo* with those of virtual FDs. Based on our methods, we demonstrated that both FD deployment methods produced similar hemodynamic results when their metal coverage was approximately equivalent. Thus, our method of virtual FD deployment was feasible with low MC FDs. Our study also confirmed that the hemodynamic changes were positively correlated with the local metal coverage of the stent at the neck and further highlighted the importance of accurately representing FDs.
